# Supplementary figures and images for: Voxel‐wise intermodal coupling analysis of two or more modalities using local covariance decomposition
Source: Hum Brain Mapp. 2022 Jun 22;43(15):4650–63. doi: 10.1002/hbm.25980 (PMC9491276; doi:10.1002/hbm.25980)

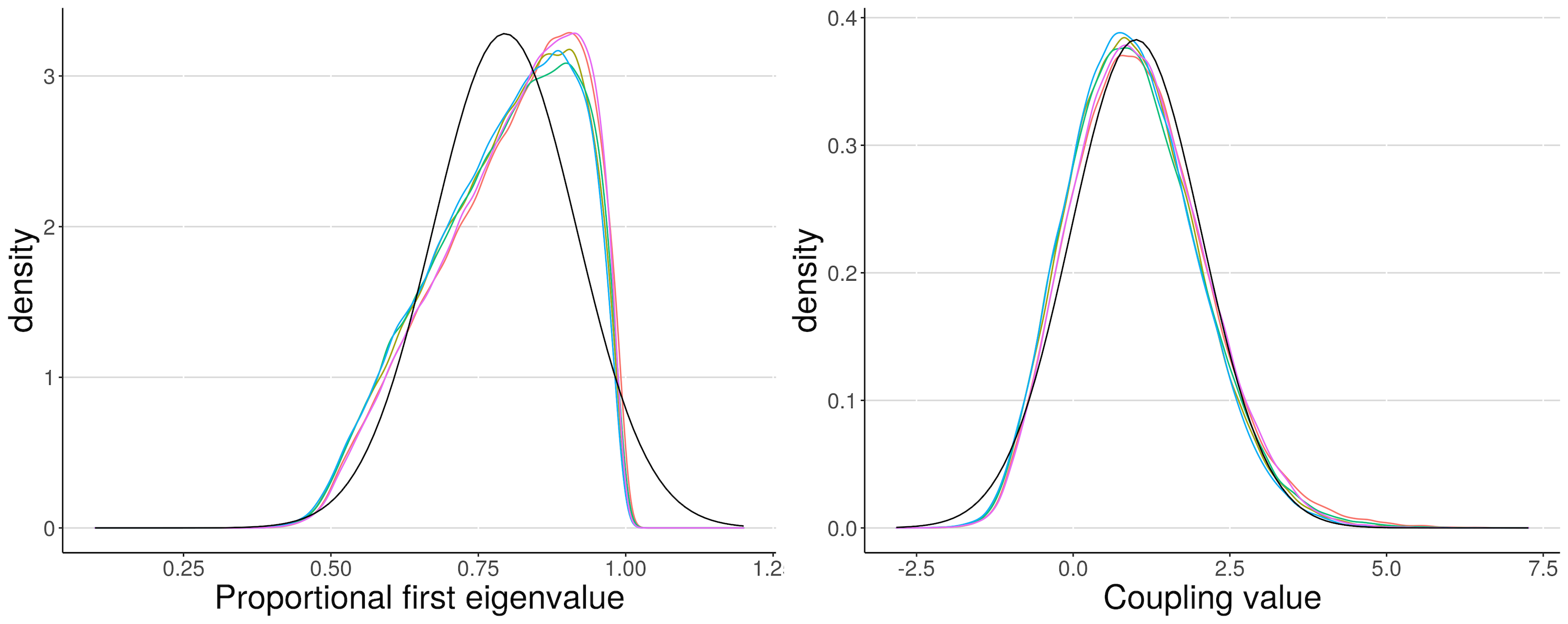

Supplement: Supplementary file 1 — Figure S1 Example of change in distribution due to the proposed transformation from proportional first eigenvalue to coupling value. Colored lines represent density plots of proportional first eigenvalues or coupling values from five randomly selected subjects. Black lines represent the corresponding normal distributions – mean and standard deviation was calculated for both proportional first eigenvalues and coupling values across the five subjects. [file HBM-43-4650-s003.tiff]

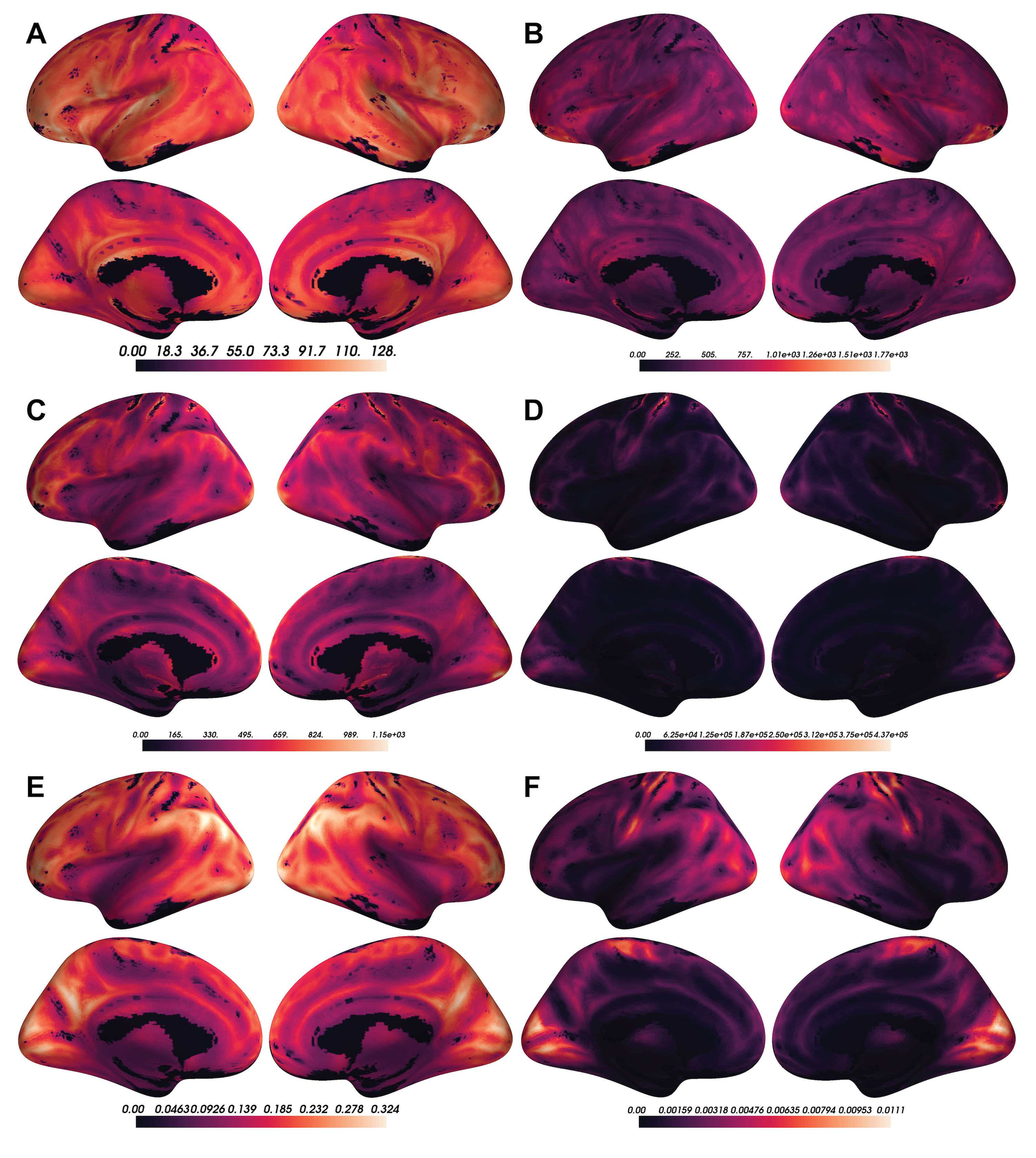

Supplement: Supplementary file 2 — Figure S2 A) Voxel‐wise means across subjects of cortical CBF. B) Voxel‐wise variances across subjects of cortical CBF. C) Voxel‐wise means across subjects of cortical ALFF. D) Voxel‐wise variances across subjects of cortical ALFF. E) Voxel‐wise means across subjects of cortical ReHo. F) Voxel‐wise variances across subjects of cortical ReHo. [file HBM-43-4650-s005.tiff]

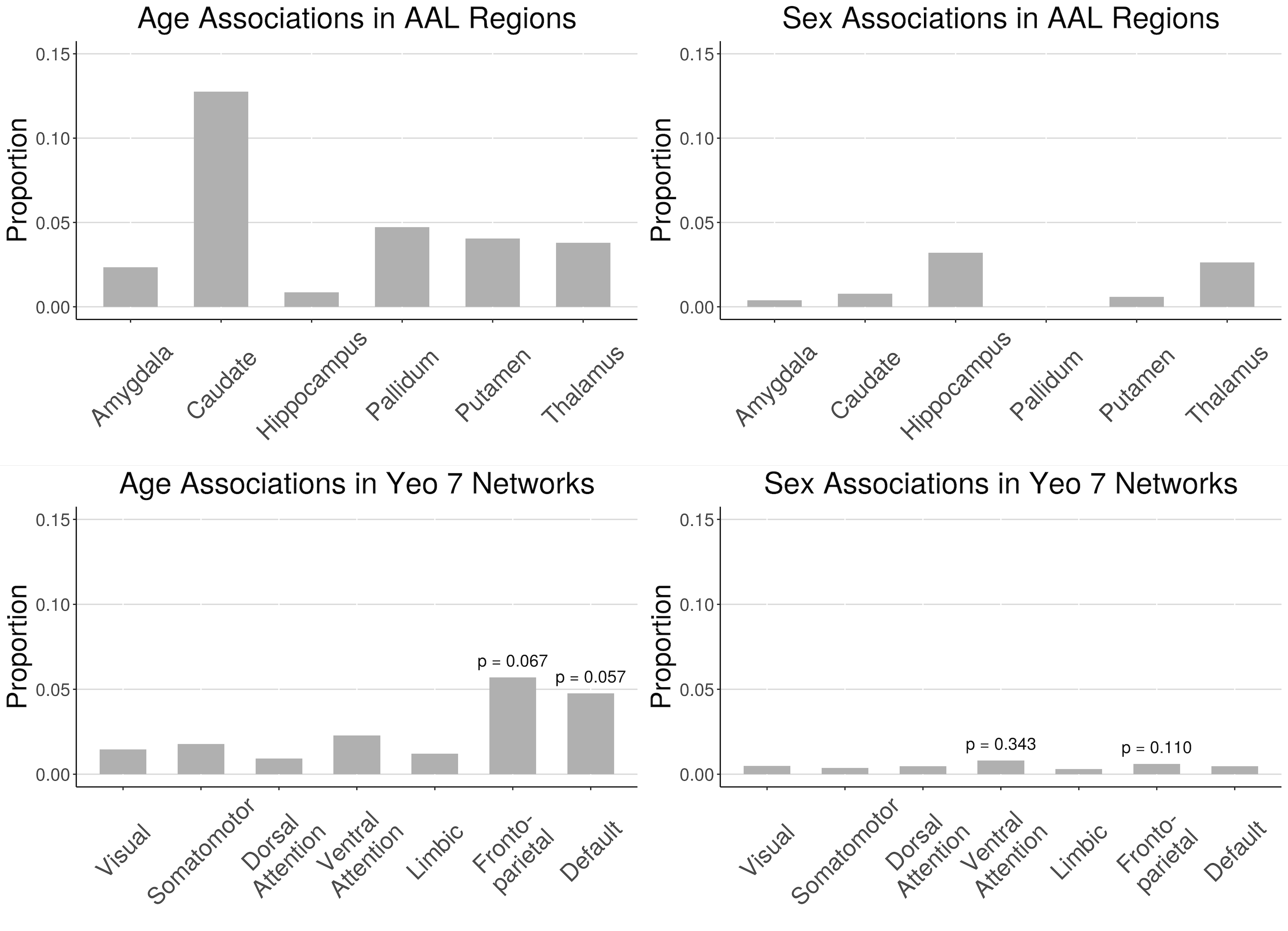

Supplement: Supplementary file 3 — Figure S3 Results from re‐analysis using FWHM of 2mm. Proportion of voxels in AAL subcortical regions and Yeo 7 cortical networks that showed significant coupling‐age and coupling‐sex associations when in‐scanner motion was included as a covariate (FDR corrected p < 0.05). Spin test was performed for all Yeo 7 networks; the two lowest p‐values are reported for each of age and sex. No p‐values were significant at alpha level of 0.05, but the lowest p‐values correspond to similar networks as analyses done with FWHM of 3mm and 5mm. [file HBM-43-4650-s001.tiff]

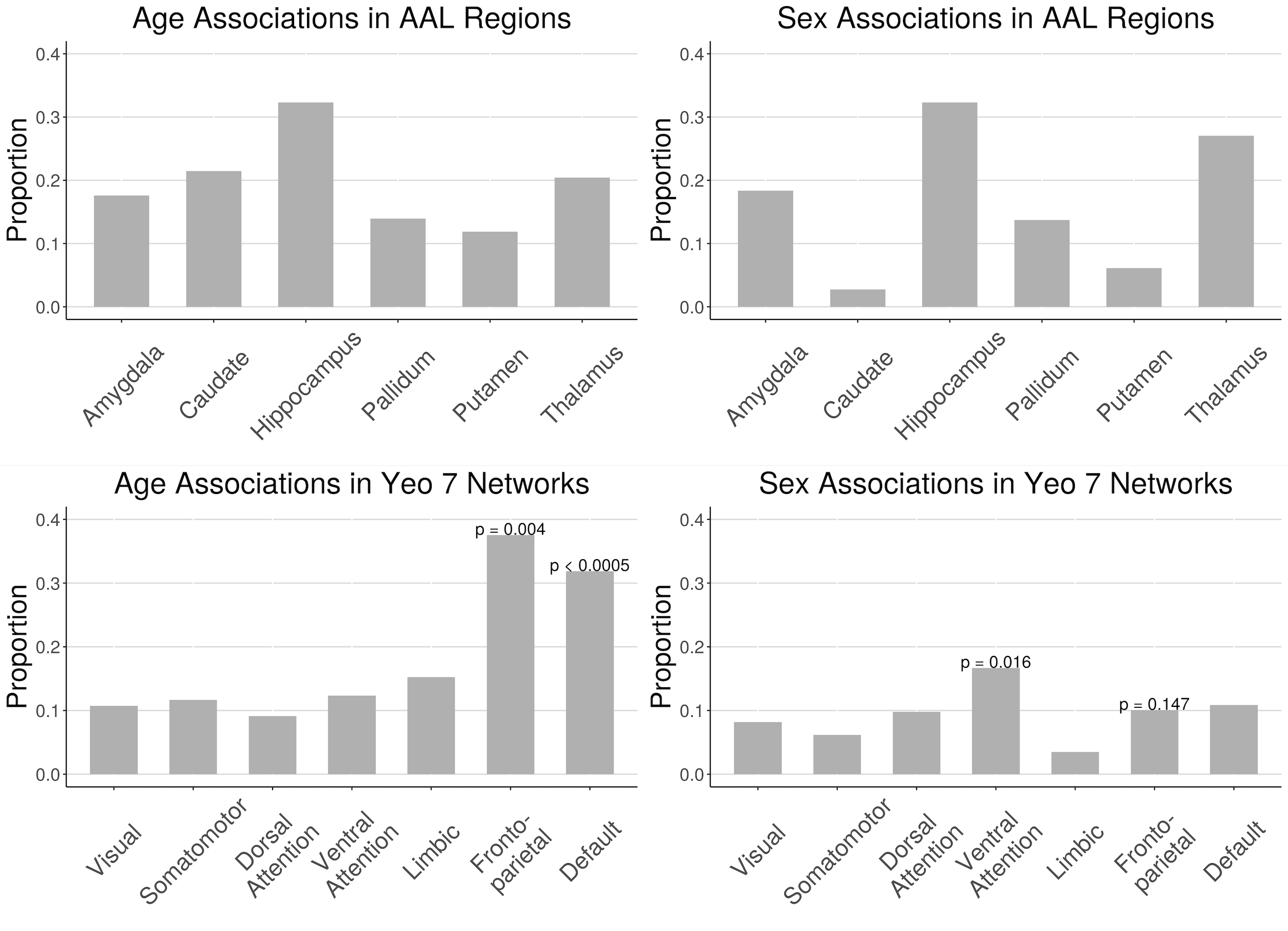

Supplement: Supplementary file 4 — Figure S4 Results from re‐analysis using FWHM of 5mm. Proportion of voxels in AAL subcortical regions and Yeo 7 cortical networks that showed significant coupling‐age and coupling‐sex associations when in‐scanner motion was included as a covariate (FDR corrected p < 0.05). Spin test was performed for all Yeo 7 networks; the two lowest p‐values are reported for each of age and sex. These p‐values correspond to similar networks as analyses done with FWHM of 2mm and 3mm. [file HBM-43-4650-s004.tiff]

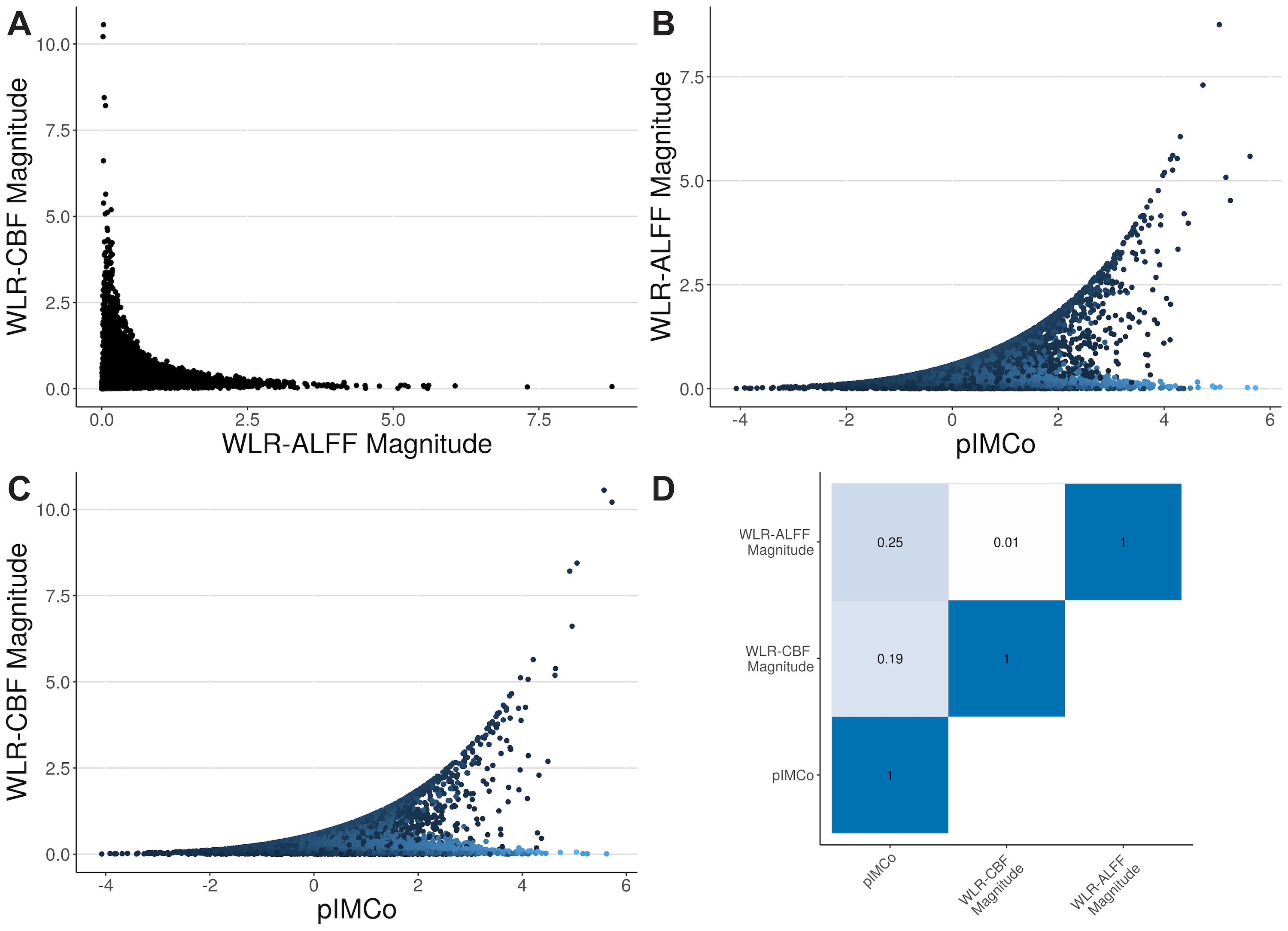

Supplement: Supplementary file 5 — Figure S5 Comparison between pIMCo coupling values and WLR‐based IMCo coupling values across 5,000 randomly sampled voxels. A) Relationship between WLR‐ALFF and WLR‐CBF coupling value magnitudes showing a strong inverse relationship. B) Relationship between pIMCo and WLR‐ALFF magnitudes. Color of points corresponds to the magnitude of the WLR‐CBF coupling value, with lighter color indicating higher magnitude. C) Relationship between pIMCo and WLR‐CBF magnitudes. Color of points corresponds to the magnitude of the WLR‐ALFF coupling value, with lighter color indicating higher magnitude. D) Pairwise non‐parametric Kendall correlations between pIMCo values, WLR‐ALFF magnitudes, and WLR‐CBF magnitudes. [file HBM-43-4650-s002.tiff]
